# Pinpointing miRNA and genes enrichment over trait-relevant tissue network in Genome-Wide Association Studies

**DOI:** 10.1186/s12920-020-00830-w

**Published:** 2020-12-28

**Authors:** Binze Li, Julian Dong, Jiaqi Yu, Yuqi Fan, Lulu Shang, Xiang Zhou, Yongsheng Bai

**Affiliations:** 1 Bellaire High School, 5100 Maple St, Bellaire, TX 77401 USA; 2 Northville High School, 45700 Six Mile Road, Northville, MI 48168 USA; 3 College Preparatory School, 6100 Broadway, Oakland, CA 94618 USA; 4 The Master’s Academy, 1500 Lukas Ln, Oviedo, FL 32765 USA; 5 Department of Biostatistics, University of Michigan, Ann Arbor, MI 48109 USA; 6 Center for Statistical Genetics, University of Michigan, Ann Arbor, MI 48109 USA; 7 Department of Biology, Eastern Michigan University, Ypsilanti, MI 48197 USA; 8 Next-Gen Intelligent Science Training, Ann Arbor, MI 48105 USA

**Keywords:** GWAS, miRNA, SNP, PBC, Variants, 3′UTR

## Abstract

**Background:**

Understanding gene regulation is important but difficult. Elucidating tissue-specific gene regulation mechanism is even more challenging and requires gene co-expression network assembled from protein–protein interaction, transcription factor and gene binding, and post-transcriptional regulation (e.g., miRNA targeting) information. The miRNA binding affinity could therefore be changed by SNP(s) located at the 3′ untranslated regions (3′UTR) of the target messenger RNA (mRNA) which miRNA(s) interacts with. Genome-wide association study (GWAS) has reported significant numbers of loci hosting SNPs associated with many traits. The goal of this study is to pinpoint GWAS functional variants located in 3′UTRs and elucidate if the genes harboring these variants along with their targeting miRNAs are associated with genetic traits relevant to certain tissues.

**Methods:**

By applying MIGWAS, CoCoNet, ANNOVAR, and DAVID bioinformatics software and utilizing the gene expression database (e.g. GTEx data) to study GWAS summary statistics for 43 traits from 28 GWAS studies, we have identified a list of miRNAs and targeted genes harboring 3′UTR variants, which could contribute to trait-relevant tissue over miRNA-target gene network.

**Results:**

Our result demonstrated that strong association between traits and tissues exists, and in particular, the Primary Biliary Cirrhosis (PBC) trait has the most significant p-value for all 180 tissues among all 43 traits used for this study. We reported SNPs located in 3′UTR regions of genes (*SFMBT2,* *ZC3HAV1*, and *UGT3A1*) targeted by miRNAs for PBC trait and its tissue association network. After employing Gene Ontology (GO) analysis for PBC trait, we have also identified a very important miRNA targeted gene over miRNA-target gene network, *PFKL*, which encodes the liver subunit of an enzyme.

**Conclusions:**

The non-coding variants identified from GWAS studies are casually assumed to be not critical to translated protein product. However, 3′ untranslated regions (3′UTRs) of genes harbor variants can often change the binding affinity of targeting miRNAs playing important roles in protein translation degree. Our study has shown that GWAS variants could play important roles on miRNA-target gene networks by contributing the association between traits and tissues. Our analysis expands our knowledge on trait-relevant tissue network and paves way for future human disease studies.

## Background

A microRNA (miRNA), a noncoding RNA which contains about 22 nucleotides, plays a significant role on the regulation of gene expression. By binding to the 3′ untranslated regions (3′UTR) of the target messenger RNA (mRNA), which transfers the genetic information from DNA to the ribosome for protein synthesis in RNA polymerase, miRNA suppresses the translation of the targeting mRNA and/or can activate gene expression under certain conditions [1]. Due to those features and miRNA’s ability to control cell growth and differentiation, people believe that there is an association between miRNA’s deficiency or excess and human diseases [2].

Single nucleotide polymorphism or SNP is a change or mutation at a single position in a DNA sequence. The development of human diseases is also affected by SNP(s) on the basis of the observation that SNPs can cause synonymous and non-synonymous changes which affects amino acid sequence or protein function [3]. However, 3′ untranslated regions (3′UTR) of genes often contain variants which can change binding affinity of targeting miRNAs so that gene expression and protein translation will be affected. It’s important to pinpoint functional important variants in 3′UTRs and elucidate if the genes harboring these variants along with their targeting miRNAs are associated with genetic traits.

In many cancers, the gene *PTEN* is known to be regulated by miRNAs through competing endogenous RNA networks [4, 5]. Such miRNAs can also have other targeted genes containing SNPs that could contribute to cancers and/or human diseases by changing DNA sequences or influencing gene expression.

Genome-wide association studies (GWAS) has identified and analyzed millions of genetic risk variants which trigger the development of complex diseases [6], and it has reported significant numbers of loci hosting SNPs associated with many traits as well [7]. The disease causativeness annotation for non-coding variants (e.g. 3′UTR variants) identified from GWAS studies and clarification of their effects on miRNAs binding are challenging. MIGWAS (miRNA–target gene networks enrichment on GWAS) is an analytic pipeline to quantitatively evaluate tissue enrichment and elucidate complex biology of the genetic traits over miRNA-target gene networks which provide resources for the genetics of human complex traits, and thus contributes to a deeper understanding of miRNA’s influence on human diseases as well as drug discovery [7, 8].

Composite likelihood-based Covariance regression Network model (CoConet) is a network method for identification of trait-relevant tissues or cell types by incorporating tissue-specific gene co-expression networks [9]. CoCoNet further understands data from GWAS by demonstrating gene co-expression sub-networks which helps predict gene-level association effect sizes on and GWAS traits and diseases. Moreover, CoConet infers trait-relevant tissue based on tissue-specific gene co-expression patterns and proves that tissue-specific gene networks underlie disease etiology [9].

ANNOVAR [10] is an efficient software tool to utilize update-to-date information to annotate genetic variants based on their functional influence. SNPs reported from large scale association studies and sequencing projects can be annotated by ANNOVAR which can be utilized to report functional score and identify variants based on SNPs’ functional influence on genes [10].

The goal of our study is to identify causative SNPs which could change the binding affinity of miRNAs and genes, which could contribute to trait-tissue relevance over miRNA-target gene network.

## Methods

We obtained summary statistics in the form of marginal z-scores for 43 traits from 28 GWAS studies [11]. These studies collect a wide range of complex traits and diseases that can be classified into six phenotype categories [12, 13]: anthropometric traits (e.g. height and BMI), hematological traits (e.g. MCHC and RBC), autoimmune diseases (e.g. CD and IBD), neurological diseases (e.g. Alzheimer's disease and Schizophrenia), metabolic traits (e.g. FG and HDL), and social traits (e.g. ever smoked and college completion). We removed SNPs within the major histocompatibility complex (MHC) region (Chr6: 25–34 Mb) following [14]. We then intersected the SNPs from all the studies and retained a common set of 622,026 SNPs for analysis. We paired the marginal z-scores from these studies with the SNP correlation matrix estimated using 503 individuals of European ancestry from the 1000 Genomes Project [15] for inference.

**Fig. 1:**
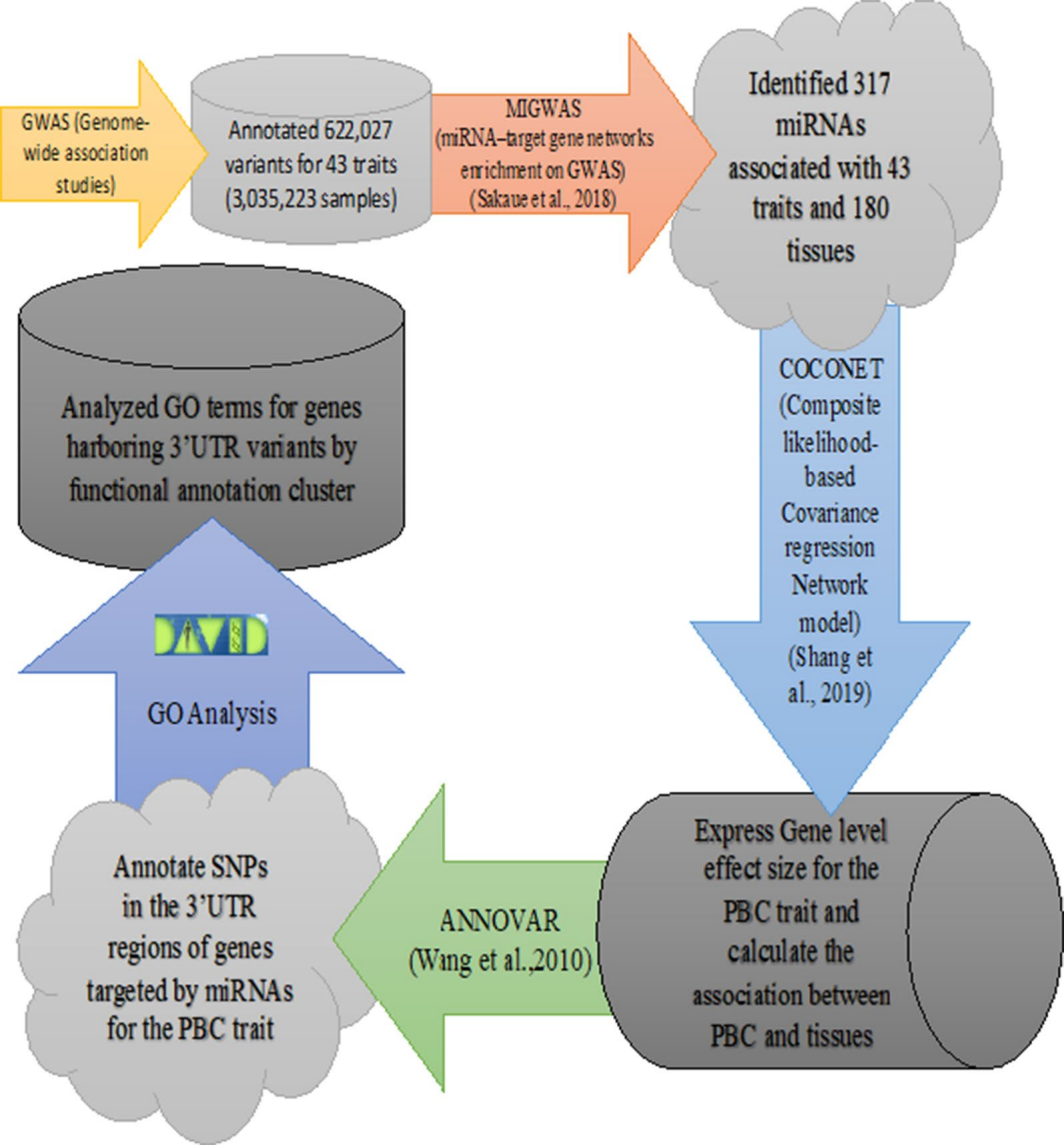
Workflow of elucidating variants and miRNAs contributed to tissue-trait association

We employed MIGWAS [7], CoCoNet [9], and ANNOVAR [10] software packages to infer trait-relevant tissues based on next-generation sequencing omics data (e.g. GTEx data [16]) and annotated the variants obtained from GWAS and harbored by miRNAs targeted genes associated with traits. We also conducted functional analysis on miRNA targeted genes harboring 3′UTR variants with significant tissue-trait association using annotation tool DAVID [17, 18] for the Primary Biliary Cirrhosis (PBC) trait (Fig. 1).

## Results

### Data acquisition

We obtained 43 traits from various published resources and classified them into six different categories with the total number of samples 3 × 10^6^ (Table 1). Details of the information for the summary statistics of 43 traits from 29 GWAS studies are provided in Additional file 1: Table S1. The table lists the phenotype name, category, abbreviation, number of individuals, reference, and downloaded websites for each of the 43 traits.

**Table 1:** Total 43 traits and number of samples adopted in the study

Phenotype	Study category	Abbreviation	# of Samples
Birth length	Anthropometric	BL	28,459
Birth weight	Anthropometric	BW	26,836
Bone density in the femoral neck	Anthropometric	FNBMD	32,961
Bone density in the lumbar spine	Anthropometric	LSBMD	31,800
Childhood obesity	Anthropometric	Child_Obes	13,848
Childhood body mass index	Anthropometric	Child_BMI	35,668
Height	Anthropometric	Height	253,288
Pubertal growth	Anthropometric	PG	13,960
Hemoglobin levels	Hematological	HB	61,155
Mean corpuscular hemoglobin concentration	Hematological	MCHC	56,475
Mean cell hemoglobin	Hematological	MCH	51,711
Mean red cell volume	Hematological	MCV	58,114
Mean platelet volume	Hematological	MPV	29,755
Packed cell volume	Hematological	PCV	53,089
Platelet count	Hematological	PLT	68,102
Red blood cell count	Hematological	RBC	53,661
Crohn’s disease	Immune	CD	20,883
Inflammatory bowel disease	Immune	IBD	34,652
*Primary biliary cirrhosis*	Immune	PBC	13,239
Rheumatoid arthritis	Immune	RA	37,681
Systemic lupus erythematosus	Immune	Lupus	14,267
Ulcerative colitis	Immune	UC	27,432
Age at menarche	Metobolic	Menarche	182,416
Coronary artery disease	Metobolic	CAD	77,210
Fasting glucose	Metobolic	FG	46,186
High-density lipoproteins	Metobolic	HDL	97,749
Heart rate	Metobolic	HR	181,171
Low-density lipoproteins	Metobolic	LDL	93,354
Total cholesterol	Metobolic	TC	100,184
Triglycerides	Metobolic	TG	94,461
Type 1 diabetes	Metobolic	T1D	26,890
Type 2 diabetes	Metobolic	T2D	60,786
Alzheimer’s disease	Neurological	Alzheimer	54,162
Autism	Neurological	Autism	10,263
Attention deficit-hyperactivity disorder	Neurological	ADD	5422
Bipolar disorder/Schizophrenia	Neurological	BIPSCZ	39,202
Bipolar disorder	Neurological	BIP	16,731
Depressive symptoms	Neurological	DS	161,460
Schizophrenia	Neurological	SCZ	70,100
College completion	Social	College	126,559
Ever smoked	Social	EverSmoked	74,053
Neuroticism	Social	Neuroticism	170,911
Years of education	Social	YE	328,917

Italic traits gave significant (p-value < 0.01) results identified by MIGWAS

### MIGWAS Results

**Fig. 2:**
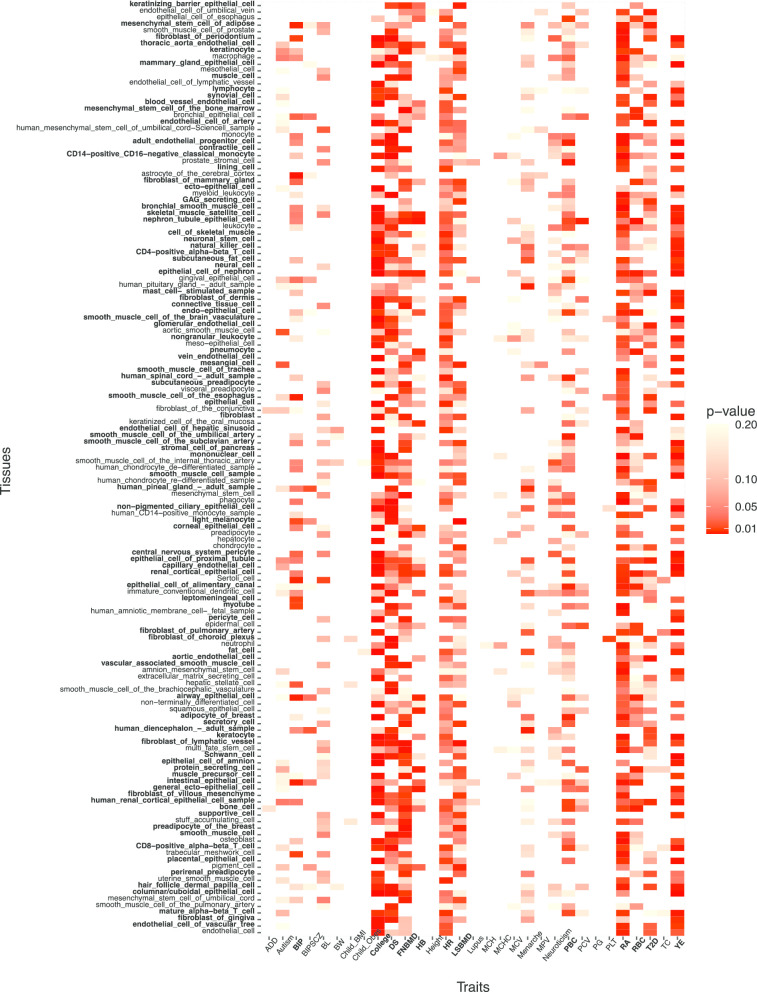
Association between tissues and traits identified by MIGWAS for 43 GWAS

We applied MIGWAS to 43 traits (total samples = 3,035,223) and identified biologically relevant tissues. In particular, we identified that 94 out of 180 tissues have significant (p-value < 0.01) association with at least two traits and that 11 traits have significant association with more than 10 tissues (Fig. 2).

**Fig. 3:**
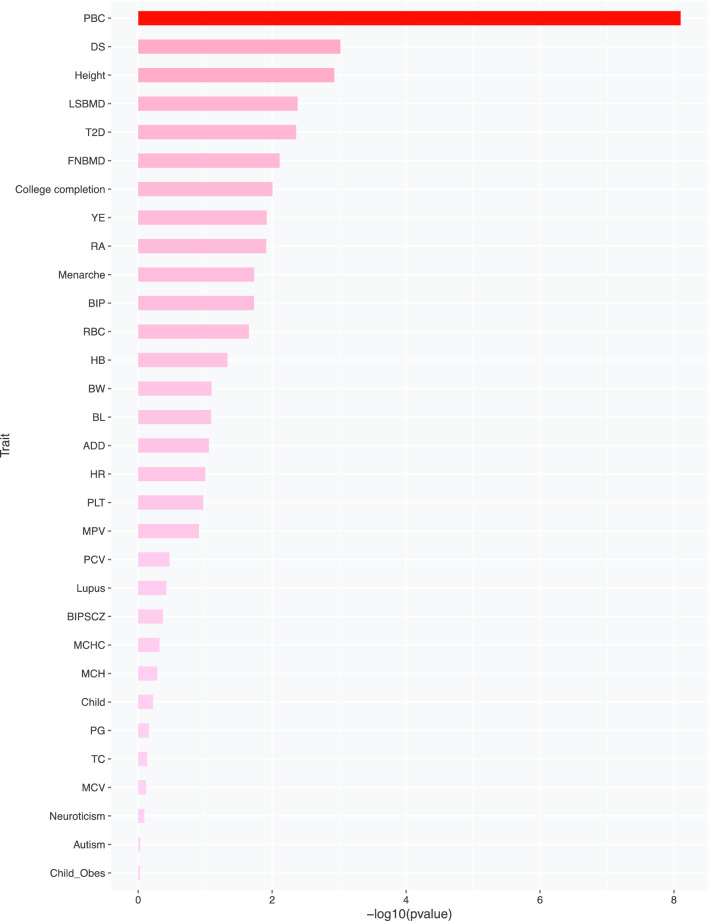
Association between traits with all tissues identified by MIGWAS for 43 GWAS

We calculated p-value association between traits and all tissues using MIGWAS for 43 GWAS. Interestingly, it reported that PBC trait has the most significant p-value for all tissues (Fig. 3). A detailed association result from MIGWAS is shown in Additional file 2: Table S2.

**Fig. 4:**
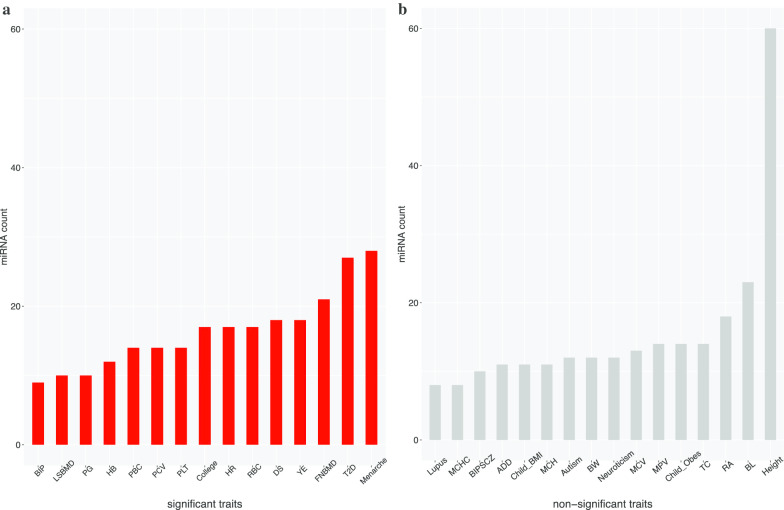
The miRNA count distribution for all reported traits by MIGWAS (**a** includes significant traits whose p-value are less than 0.01 in at least one tissue, while **b** includes non-significant traits

We have identified total 332 miRNAs as candidate biomarkers of all traits. The miRNA distribution statistics for all reported traits by MIGWAS is shown in Fig. 4. We found that there are no miRNAs identified through MIGWAS overlapping across significant tissue-traits. We divided 31 traits into two groups that the significant trait group has 15 traits and the non-significant trait group has 16 traits. If the p-value of the trait is less than 0.01 in at least one tissue, it is considered as a significant trait. The non-significant trait *Height* has 60 miRNAs which is the most among all 31 traits. A list of detailed gene-miRNA pairs reported for all traits is shown in Additional file 3: Table S3.

### CoCoNet output

**Fig. 5:**
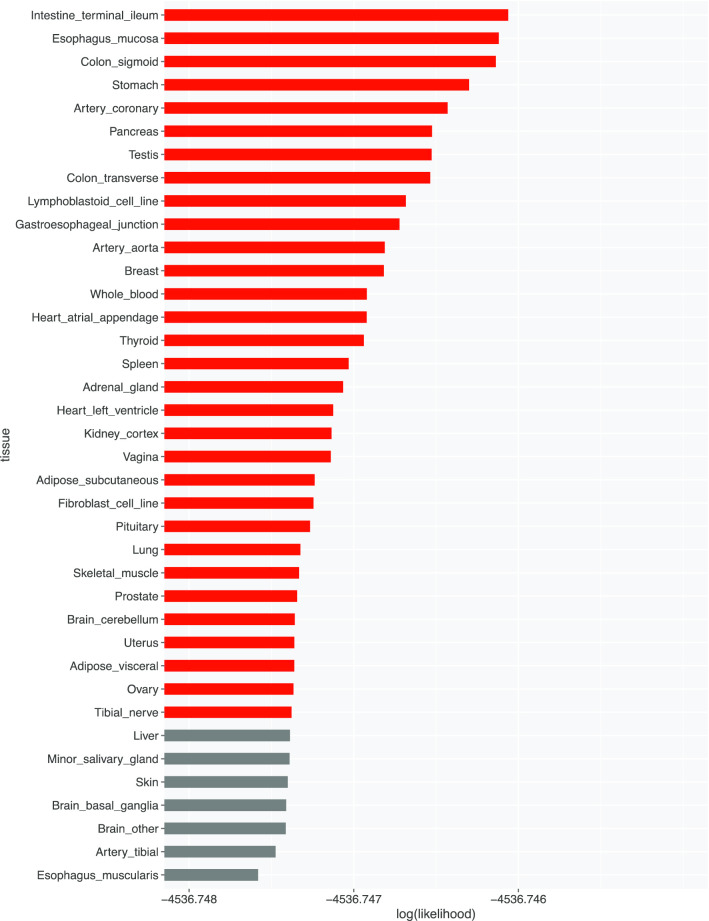
Likelihood values between PBC trait and tissues (red ones have greater loglikelihood based on − 4536.7474 (the log(likelihood) for PBC))

We took candidate target genes identified by MIGWAS to retrieve Gene Level Effective Size of each gene (Ensemble gene ID) for the PBC trait. We used CoCoNet to calculate the association between traits and tissues in terms of log likelihood (Fig. 5).

### ANNOVAR annotation

We examined the PBC trait showing the smallest association p-value reported by MIGWAS to see if there are any SNPs located in 3′UTR regions of genes targeted by miRNAs. To do so, we took the SNP list for the PBC trait and converted them to ANNOVAR variant annotated input format with ANNOVAR package. We obtained 9,890 annotated variants (5279 genes) exclusively located in 3′UTR region of these genes (Additional file 4: Table S4).

We crossed the 55 genes reported by MIGWAS with 5279 genes annotated by ANNOVAR and obtained 13 genes in common. The detailed information about these 13 genes is shown in Table 2.

**Table 2:** Annotation information for 13 genes shared by MIGWAS and ANNOVAR

Chr	Start	End	Ref	Alt	Func.refGene	Gene.refGene	Targeting.MiRNA
chr5	35,952,140	35,952,140	C	T	UTR3	*UGT3A1*	*hsa-mir-1207*
chr5	35,953,169	35,953,169	G	A	UTR3	*UGT3A1*	*hsa-mir-1207*
chr5	35,953,320	35,953,320	C	T	UTR3	*UGT3A1*	*hsa-mir-1207*
chr5	35,954,143	35,954,143	C	T	UTR3	*UGT3A1*	*hsa-mir-1207*
chr1	23,755,513	23,755,513	T	C	UTR3	*ASAP3*	*hsa-mir-1207*
chr1	23,755,716	23,755,716	T	C	UTR3	*ASAP3*	*hsa-mir-1207*
chr1	23,756,155	23,756,155	T	C	UTR3	*ASAP3*	*hsa-mir-1207*
chr12	42,475,888	42,475,888	G	A	UTR3	*GXYLT1*	*hsa-mir-1205*
chr12	42,476,613	42,476,613	A	C	UTR3	*GXYLT1*	*hsa-mir-1205*
chr12	42,478,556	42,478,556	A	C	UTR3	*GXYLT1*	*hsa-mir-1205*
chr12	42,479,450	42,479,450	A	G	UTR3	*GXYLT1*	*hsa-mir-1205*
chr12	42,481,545	42,481,545	T	C	UTR3	*GXYLT1*	*hsa-mir-1205*
chr21	34,730,954	34,730,954	G	A	UTR3	*IFNAR1*	*hsa-mir-1976*
chr3	119,243,549	119,243,549	G	A	UTR3	*CD80*	*hsa-mir-16-2*
chr3	119,243,934	119,243,934	G	A	UTR3	*CD80*	*hsa-mir-16-2*
chr3	119,244,421	119,244,421	A	C	UTR3	*CD80*	*hsa-mir-16-2*
chr22	39,778,167	39,778,167	A	G	UTR3	*SYNGR1*	*hsa-mir-4728*
chr22	39,778,327	39,778,327	T	C	UTR3	*SYNGR1*	*hsa-mir-4728*
chr22	39,778,419	39,778,419	T	C	UTR3	*SYNGR1*	*hsa-mir-4728*
chr22	39,780,961	39,780,961	C	T	UTR3	*SYNGR1*	*hsa-mir-4728*
chr22	20,796,175	20,796,175	T	C	UTR3	*KLHL22*	*hsa-mir-1207*
chr11	111,223,111	111,223,111	A	G	UTR3	*POU2AF1*	*hsa-mir-4728*
chr10	7,202,028	7,202,028	C	T	UTR3	*SFMBT2*	*hsa-mir-1207*
chr10	7,202,931	7,202,931	C	T	UTR3	*SFMBT2*	*hsa-mir-1207*
chr20	1,545,468	1,545,468	T	C	UTR3	*SIRPB1*	*hsa-mir-1207*
chr20	1,545,617	1,545,617	T	C	UTR3	*SIRPB1*	*hsa-mir-1207*
chr20	1,609,952	1,609,952	C	T	UTR3	*SIRPG*	*hsa-mir-1207*
chr20	1,610,201	1,610,201	C	T	UTR3	*SIRPG*	*hsa-mir-1207*
chr7	138,729,795	138,729,795	A	C	UTR3	*ZC3HAV1*	*hsa-mir-1206*
chr7	138,730,025	138,730,025	T	C	UTR3	*ZC3HAV1*	*hsa-mir-1206*
chr7	138,730,361	138,730,361	T	C	UTR3	*ZC3HAV1*	*hsa-mir-1206*
chr7	138,731,398	138,731,398	T	C	UTR3	*ZC3HAV1*	*hsa-mir-1206*
chr7	138,732,107	138,732,107	C	T	UTR3	*ZC3HAV1*	*hsa-mir-1206*
chr19	37,674,259	37,674,259	G	A	UTR3	*ZNF585B*	*hsa-mir-1237*

### PTEN associated variant analysis

Since PBC is a liver-related trait, we searched the TCGA LIHC dataset [19] and checked anti-correlated pairs for *PTEN* targeted miRNA: *hsa-mir-590*. We then used *hsa-mir-590* to identify its targeted genes in the anti-correlated pair list and obtained 22 additional target genes. We then identified SNPs using UCSC Genome Browser for ten genes including *PTEN* with ClinVar variant information and reported the result in a new Additional file 5: Table S5.

### Functional analysis

Gene Ontology (GO) analysis on 55 miRNA targeted genes harboring 3′UTR variants with significant tissue-trait association for PBC trait only showed that *PFKL* is located in a set of genes with term Acetylation (p-value < 0.05). This gene encodes the liver (L) subunit of an enzyme that catalyzes the conversion of d-fructose 6-phosphate to d -fructose 1,6-bisphosphate, which is a key step in glucose metabolism (glycolysis) [20].

The list of genes and their GO terms identified as the top cluster by DAVID is shown in Table 3.

**Table 3:** Genes and GO terms in the top cluster for 55 genes identified by DAVID

Genes	Term	Category	p-value
*PFKL, ZC3HAV1, PKN1, SYNGR1, STAT6, LRP1, CDKN2A, CLIC4, MAPT, KLHL22, MYH14, UBLCP1, CARM1, TNFAIP3, ARHGDIB, PRPF40A*	Acetylation	UP_KEYWORDS	0.02307187
*PFKL, ZC3HAV1, KIF17, DTX3, PKN1, ASAP3, CASC3, RALGDS, STAT6, LRP1, CDKN2A, CLIC4, MAPT, KLHL22, SPIB, CARM1, TNFAIP3, KIF21B, ARHGDIB*	Cytoplasm	UP_KEYWORDS	0.05299595
*PFKL, KIF17, PKN1, CASC3, RALGDS, STAT6, CDKN2A, RASGRF1, CLIC4, MAPT, MYH14, CARM1, TNFAIP3, ARHGDIB*	GO:0005829~cytosol	GOTERM_CC_DIRECT	0.15378123
*PFKL, ZC3HAV1, RRP7A, DTX3, PKN1, ASAP3, STAT6, LRP1, CDKN2A, CLIC4, MAPT, KLHL22, SPIB, CARM1, TNFAIP3, KIF21B, ARHGDIB, PRPF40A*	GO:0005737~cytoplasm	GOTERM_CC_DIRECT	0.33828457

We have also conducted a GO analysis for 22 genes targeted by *hsa-mir-590* with target relationship of *PTEN*. Some enrichment analysis results are reported in Table 4. The detailed DAVID results are reported in Additional file 6: Table S6 and Additional file 7: Table S7.

**Table 4:** Genes and GO terms targeted by *hsa-mir-590* with target relationship of *PTEN*

Genes	Term	Category	p-value
*RNF180, CYP2U1, ARIH1, AGBL5, ZHX1, BMPR2, NR3C1, RHOU, RNF32, FOXP1, PGGT1B*	Metal-binding	UP_KEYWORDS	0.001554879
*RNF180, ARIH1, AGBL5, ZHX1, NR3C1, RNF32, FOXP1, PGGT1B*	Zinc	UP_KEYWORDS	0.006809626
*CSNK1A1, IRAK1, ARIH1, POMGNT1, FLT1, BMPR2, UBE2W, PGGT1B*	Transferase	UP_KEYWORDS	0.00109919
*CSNK1A1, RHOJ, IRAK1, FLT1, BMPR2, UBE2W, RHOU*	Nucleotide-binding	UP_KEYWORDS	0.007386586

## Discussions

In our study, we used several cutting-edge bioinformatics tools (MIGWAS, CoCoNet and ANNOVAR) to dissecting SNPs reported for 43 traits from GWAS. The MIGWAS reported miRNA enrichment over target gene network for most (31) of traits. The CoCoNet was adopted to analyze the PBC trait for its significant tissue association over gene co-expression network. The ANNOVAR tool was employed to annotate 3′UTR variants class harbored by a list of miRNA target genes associated with Primary Biliary Cirrhosis (PBC) traits.

Although it may seem like the tools are interrelated and are three separate experiments, they are indeed connected. We first applied the tool MIGWAS to 43 traits and identified significant biologically relevant tissues. When the p-value association between traits and all tissues were calculated by using MIGWAS for 43 GWAS, we found that the PBC trait had the most significant p-value for all tissues. The CoCoNet experiment then utilized some of the results produced by MIGWAS to provide an output. Specifically, for the retrieved PBC trait result from the MIGWAS experiment, we used CoCoNet to analyze the tissues association over gene co-expression network. Again, we used the PBC trait result reported by MIGWAS in another tool: ANNOVAR. We analyzed the PBC trait for SNPs in 3′UTR regions by converting the SNP list into ANNOVAR variant annotated input, and we retrieved 5279 genes located in 3′UTR regions. We then crossed the 55 genes from MIGWAS with the 5279 genes from ANNOVAR and found 13 genes in common. The experiments might seem unrelated, but the results from MIGWAS analyzed by both CoCoNet and ANNOVAR shows that the 3 tools are indeed connected and are not interrelated.

In a previous study, the research found a strong association between PBC and rs231725 [21]. rs231725 is a SNP in the 3′ flanking region of *CTLA4*, a gene which has an impact on the risk of PBC [21]. Indeed, *PTEN* is associated with 9 traits (Crohn's disease [22], Inflammatory bowel disease [23], Rheumatoid arthritis [24], Systemic lupus erythematosus [25], Ulcerative colitis [23], Coronary artery disease [26], Type 2 diabetes [27], Alzheimer's disease [28], Autism [29]) out of our analyzed 43 traits.

Total 12 traits (Alzheimer’s disease, Coronary artery disease, Crohn’s disease, Ever Smoked, Fasting glucose, High density lipoproteins, Inflammatory bowel disease, Low density lipoproteins, Schizophrenia, Type 1 diabetes, Triglycerides, Ulcerative colitis) do not have MIGWAS association results reported in the analysis. It seems that liver tissue does not appear to have the smallest likelihood with PBC based on the total genes in human genome. We also ran CoCoNet with the targeted gene set of miRNAs enriched over the target network for PBC trait only to calculate loglikelihood, and the result stays the same.

The transcript expression of *PFKL* has the highest RPKM (Reads Per Kilobase of transcript, per Million mapped reads) in kidney samples in the RNA-seq which was performed from 4 human individuals in order to determine tissue-specificity of all protein-coding genes [30].

*PFKL* contains a transcript variant *ATP-dependent 6-phosphofructokinase, liver type isoform a* which represents the longer transcript and encodes the shorter isoform (a). The CD-Search shows the protein classification of this transcript variant, which is *Eukaryotic_PFK domain-containing protein*, and finds a specific hit, *Eukaryotic_PFK*, which shows a high confidence association between the query sequence and a domain model. Phosphofructokinase (PFK) is a key regulatory enzyme that that phosphorylates fructose 6-phosphate in glycolysis. It belongs to the PFK family, evolving from the bacterial PFKs by gene duplication and fusion events and then exhibiting complex behavior. PFK family also includes ATP-dependent phosphofructokinases (allosteric homotetramers) and pyrophosphate (PPi)-dependent phosphofructokinases (mostly dimeric and nonallosteric homotetramers). In addition, protein sequences of Opisthokonta are in the multiple sequence alignment in the *Eukaryotic_PFK* [30].

We found 13 genes, *UGT3A, SYNGR, ASAP3, CD80, GXYLT1, IFNAR, KLHL22, POU2AF1, SFMBT2, SIRPB1, SIRPG, ZC3HAV1* and *ZNF585B* which are in common in the gene list applied from MIGWAS and ANNOVAR.

*ASAP3* encodes a member of a subfamily of ADP-ribosylation factor(Arf) GTPase-activating proteins that its expression level affects cell proliferation and migration, and it called up-related in liver cancer 1 [31]. *DDEFL1* (cloned *ASAP3*) is unregulated in hepatocellular carcinoma (HCC) through microarray analysis. The studies [32] showed that *DDEFL1* had increased expression and had overexpression which increased colony formation in *NIH3T3* cells and human hepatoma cell lines [32].

In the transcript expression of *UGT3A1*, liver samples have the second highest RPKM among 27 different tissues [33].

*SFMBT2* are polycomb group proteins that bind to methylated lysins in histone tails. The formation of transcription-resistant higher-order chromatin structures at target genes are induced by it so that this gene can repress transcription [34]. *SFMBT2* was cloned and designated as *KIAA1617* by Nagase et al. [35], who obtained clones from fetal brain cDNA library and then sequenced them [35]. Kuzmin et al. [36] reported that *SFMBT2* interacted with the *YY1* (600013) transcription when *SFMBT2* was expressed from the paternal allele in mouse blastocysts and in mouse embryonic tissues early in development, and later during mouse embryonic development [36].

*ZC3HAV1* encodes a zinc finger protein that can prevent replication certain viruses and inhibit viral gene expression by targeting and eliminating viral mRNAs in the cytoplasm [37]. Yu et al. [38] identified *ZC3HAV1* and called it ZAP in their analysis of gene expressed in human fetal liver [38]. Northern blot analysis of rat Zap that was cloned by Gao et al. [39] showed that mRNA was highly expressed in kidney and liver [39].

## Conclusions

Our study tried to understand GWAS data through identifying candidate miRNA-gene pairs over miRNA-target gene network. Using several cutting-edge bioinformatical tools and databases and adopting visualization, we assessed trait-tissue relevance based on tissue-specific gene co-expression information including protein–protein interaction and transcription factor binding evidence. We annotated 3′UTR variants harbored by genes targeted by miRNAs expressed in tissues significantly associated with PBC trait. Our study provides evidence that the association between tissues and traits could be affected by the 3′UTR variants of genes which change binding affinity of targeting miRNAs. The analysis emphasizes the influence of variants on genetic traits and miRNA-targeting gene networks, and thus could contribute to additional studies and detections on human diseases. We think our study provided a valuable approach for elucidating 3′UTR variants which could contribute to genetic traits with tissue relevance in the context of miRNA influential human diseases.

## Supplementary information

**Additional file 1: Table S1.** Details of the summary statistics of 43 traits from 29 GWAS studies.

**Additional file 2: Table S2.** Tissue association result from MIGWAS.

**Additional file 3: Table S3.** Gene-miRNA target pairs identified by MIGWAS for each trait.

**Additional file 4: Table S4.** Identified 9890 annotated variants exclusively located in 3′UTR region of 5279 genes.

**Additional file 5: Table S5.** Identified SNPs for ten genes with ClinVar variant information.

**Additional file 6: Table S6.** Genes and GO terms in the top cluster for 55 genes with significant tissue and PBC trait association identified by DAVID.

**Additional file 7: Table S7.** Genes and GO terms in the top cluster for *hsa-mir-590* with target relationship of *PTEN.*

## Abbreviations

miRNA: Micro ribonucleic acid
RNA: Ribonucleic acid
mRNA: Messenger ribonucleic acid
DNA: Deoxyribonucleic acid
SNP: Single-nucleotide polymorphism
3′UTR: Three prime untranslated region
GWAS: Genome-wide association study
MIGWAS: MiRNA–target gene networks enrichment on GWAS
CoConet: Composite likelihood-based Covariance regression Network model
GO: Gene Ontology
PFK: Phosphofructokinase
TCGA: The Cancer Genome Atlas
LIHC: Liver hepatocellular carcinoma
